# Exploring Changes in Thalamus Metabolites as Diagnostic Biomarkers in Idiopathic Generalised Epilepsy Patients Using Magnetic Resonance Spectroscopy

**DOI:** 10.21315/mjms2020.27.1.8

**Published:** 2020-02-27

**Authors:** Razzagh Abedi-Firouzjah, Ayoob Rostamzadeh, Amin Banaei, Mohsen Shafiee, Zafar Masoumi Moghaddam, Hassan Vafapour

**Affiliations:** 1 Cellular and Molecular Research Center, Yasuj University of Medical Sciences, Yasuj, Iran; 2 Department of Anatomical Sciences, Faculty of Medicine, Shahrekord University of Medical Sciences, Shahrekord, Iran; 3 Department of Medical Physics, Faculty of Medical Sciences, Tarbiat Modares University, Tehran, Iran; 4 Department of Radiology, Faculty of Paramedical Sciences, AJA University of Medical Sciences, Tehran, Iran; 5 Social Determinants of Health Research Center, Yasuj University of Medical Sciences, Yasuj, Iran

**Keywords:** spectroscopy, MRS, MRI, epilepsy

## Abstract

**Introduction:**

Idiopathic generalised epilepsy (IGE) refers to a group of epilepsies resulting from the activation of neurons in the whole brain. This study aimed to evaluate the metabolite changes in thalamus as diagnostic biomarkers in IGE patients compared to healthy individuals using magnetic resonance spectroscopy (MRS) technique.

**Methods:**

The MRS was performed on 35 IGE patients (26 women and 11 men) with average age of 32 (ranged from 18 to 43) and 35 healthy individuals (13 women and 22 men) with average age of 31 (ranged from 21 to 50) as the control group. The levels of N-acetylaspartate (NAA), creatine (Cr) and choline (Cho) were measured using MRS. The NAA/Cr and NAA/Cho ratios were calculated for all participants. These values were statistically compared using *t*-test between the groups.

**Results:**

The NAA had significant lower values in IGE patients, 9.6 (SD = 0.8) and 9.9 (SD = 0.7) for right and left thalamus, respectively, compared to 10.9 (SD = 0.9) and 10.7 (SD = 0.9) in control group. The Cr values in the left side of thalamus were significantly higher in IGE patients (6.7 [SD = 0.8] versus 5.8 [SD = 0.5]); however, there was no difference in right thalamus. Measurements showed no difference for amounts of Cho between the groups in both sides of thalamus. The NAA/Cr ratio was 1.48 (SD = 0.14) and 1.48 (SD = 0.16) for right and left thalamus, respectively, in IGE patients in comparison with 1.83 (SD = 0.2) and 1.86 (SD = 0.26) in controls. There was no meaningful variation between the NAA/Cho ratio of the right and left thalamus among the groups.

**Conclusion:**

Thalamic NAA, Cr and NAA/Cr ratio values in IGE patients showed statistical differences compared to healthy individuals. Evaluating metabolites variations in thalamus using MRS is suggested for differentiating IGE patients from healthy individuals.

## Introduction

A seizure is a process that occurs as a result of abnormal, excessive and synchronous electrical depolarisation of a group of central neurons in brain. The diagnosis of epilepsy is made when seizure attacks are repeated due to a chronic background process (1).

Idiopathic generalised epilepsy (IGE) refers to a group of epilepsies resulting from the activation of neurons in the whole-brain (2). This type of epilepsy shows more side effects due to its resistance to medical treatments. The diagnosis of IGE is based on strict clinical guidelines and electroencephalogram (EEG) as proposed by the International League Against Epilepsy (ILAE). IGE is more common in the first or second decade of life; although, it may be present in any ages (3, 4). The patients suffer from epilepsy, might return to normal life situation after surgical operations on the seizure foci resistant against drug treatment. Therefore, the major part of efforts in epilepsy diagnosis is focusing on seizure foci detection.

Magnetic resonance imaging (MRI) has become one of the useful diagnostic techniques for high-resolution structural imaging in epilepsy (5). Magnetic resonance spectroscopy (MRS) is a non-invasive imaging strategy to provide metabolic information from different body tissues, especially in human brain. MRS is a diagnostic technique which can assess the neurochemical changes in a given brain region of interest. The detection of the chemical molecules has been valuable for understanding the presence of neuronal elements, cell proliferation and degradation. MRI and MRS rely on the same physics and interactions to gather magnetic resonance (MR) signals; however, they have some differences in data processing, data displaying and interpretation. Data are presented as spectrum plots in MRS (6). Various metabolites can be detected in these spectrums. Three major peaks characterise long-echo time MRS spectra: N-acetylaspartate (NAA) (marker of neuronal and axonal viability, and density), creatin (Cr) (used as internal reference, since it is the most stable cerebral metabolite) and choline (Cho) (reflecting cellular proliferation) (7).

The thalamus plays a central role in cortical synchronisation (8) [for a review, see (9)]. The function of thalamus is vital for sensory, motor and cognitive functions, therefore, it has been the subject of many neurological studies. Experimental animal studies of generalised epilepsy confirmed that the thalamo-cortical circuitry is involved in spike and wave discharge generation (10). According to these studies, a dysfunction in reticular thalamic neurons may alter the electro-responsiveness of the developing thalamo-cortical system and cause a persistent state of abnormal excitation (9). Study of thalamus provides a valuable information regarding the role of thalamus in generalised epilepsy and electrophysiological data as the major mechanism of IGE (11, 12).

The neurochemical abnormalities reflect thalamo-cortical dysfunction reported in Bernasconi et al. (11) study, therefore, measuring the amount of thalamus metabolites such as NAA, Cho and Cr can be a way of early diagnosis of epilepsy (13).

MRS can assist some pathophysiological approaches and seizure generating mechanisms and also play a remarkable role in applied research studies aimed at examining the early seizure disorder processes (7, 14). In addition, MRS able to detect metabolic disorders which are invisible to a typical MRI procedure.

Previous studies (11, 12, 15–17) focused on the changes of NAA and NAA/Cr values of thalamus in IGE patient, therefore, it was proposed to assess the thalamus Cr, Cho and NAA/Cho values in IGE patients in future researches. Due to the lack of human studies assessing the thalamus metabolites changes in IGE patients, the aim of this study was to evaluate and compare the thalamus metabolites in IGE sufferers using MRS technique in patients and healthy groups.

## Methods

### Subjects

The present cross-sectional study was carried out (between June 2018 and February 2019) on 70 individuals; 35 IGE patients whose illness had been established based on the seizure history and the EEG findings. The sample size was determined based on previous studies (11, 13). The average age of patients and healthy (control) groups were 32 (ranged from 18 to 43) and 31 (ranged from 21 to 50), respectively. The patient’s demographic information has been illustrated in Table 1. The patients were randomly selected from the database of patients referred to the epilepsy centre of Shohadey-e-Tajrish Hospital (Tehran, Iran). The patients and control groups had a normal morphologic cranial MRI images. None of the patients had a seizure 24 h before MR scan. All individuals were right-handed.

All of the patients consumed their anti-epileptic drug during MRS investigation which were on various pharmacotherapy programmes: valproate (13 patients); a combination of valproate and lamotrigine (9 patients); a combination of valproate, lamotrigine and carbamazepine (7 patients); carbamazepine (6 patients) and their disease duration were 5 years, 1 year and 7 months, 2 years and 4 months, and 12 years, respectively. The patients received their prescribed medication process as in the past. There was not any history of mental or neurological problems in healthy individuals. Informed consent was obtained from all participants after the procedures were fully explained and the study received the approval of the national ethics committee with the registration number of IR.YUMS.REC.1396.148. Diagnostic and therapeutic methods had not any changes in patients group compared to regular methods in our study. MRI and MRS scans had not been reported to have any side effects (11, 13).

### MRI Protocol

A Siemens Avanto MRI machine (Siemens Healthineers, Germany) with the magnetic field power of 1.5-T was used along with Syngo computer software (Siemens Healthineers, Germany) to process the data. In an ideal situation, this machine has a field consistency of 1 ppm for 50 cm and magnetic stability of 0.1 ppm. An 8-channel MRI coil was utilised for transmitting and receiving the MR electromagnetic signals.

To minimise the positioning variations of the head, patients were positioned by the same operator using the orbito-meatal line as a landmark. Incidence of possible seizure during the scan was evaluated based on the patient reports and under the continuous supervision of a physician.

The routine protocols including T1-weighted, T2-weighted and T2-trim were used to create coronal, sagittal and axial images. The MRI protocol consisted of axial T2-weighted fast spin echo images [time of echo (TE) = 56 ms, time of repetition (TR) = 2500 ms and field of view (FOV) = 24 cm with 3 mm thickness] and axial T1-weighted three dimensional spoiled gradient recalled acquisition in the steady state (GRASS) images (TE = 7 ms, TR = 23 ms, flip angle (FA) = 50°, FOV = 24 cm with 124–156 slice partitions of 1 mm thickness and two number of excitations (NEX). T2-tirm dark fluid images with TI = 2027 ms, TR = 6000 ms, TE = 88 ms and FA = 150° were obtained. The voxels of interest (VOI) were adjusted to individual anatomy on images to cover the right thalamus, right cerebellum, prefrontal cortex and occipital cortex based on a protocol proposed in a previous study by Savic et al. (18).

### MRS Protocol

**Figure 1:**
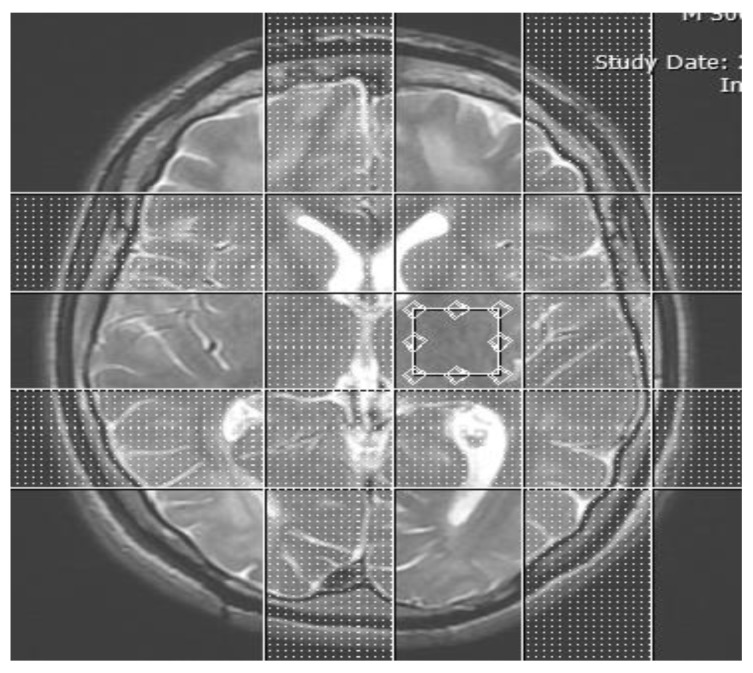
Placement of the VOI on the left thalamus

MRS with the point-resolved spectroscopy-chemical shift imaging (PRESS-CSI) technique was performed as following parameters: VOI = 3.35 mm × 2.8 mm × 2.8 mm, NEX = 2, number of repetition (NR) = 15, scan time = 6 min + 28 s, TE = 99 ms, TR = 4900 ms, TI = 2027 ms and total examination time = 44 min. Raw data and images were stored on the MRI system computer. These data were transferred to another computer for subsequent analysis. These routine MRI images were used as a positioning matrix for measuring spectrogram and for determination of the VOI positions. In Figure 1, the adjusted VOI position of left thalamus was shown.

The magnetic field homogenisation was done manually at the beginning of every protocol. Water suppression pulses were applied automatically and the pre-saturation bands were placed around the VOI area in order to remove fat and extraneous signals from the tissues around the VOI. The obtained spectral data was processed by the computer software and the results were evaluated based on the area under the curve of metabolite peaks. The metabolite concentrations in absolute units of mmol/L (mM) for the NAA, Cr and Cho were measured based on the MRS spectrum analysis. The metabolite concentrations values were analysed using the free nuclear magnetic resonance-spectra calculation using operators (NMR-SCOPE) software (Institute of Scientific Instruments, Czech Republic). This software has the ability to analyse the peaks of all metabolites (10).

Post-processing of the raw MRS data for obtaining the 32×32 profiles, followed by a mild Gaussian k-space filter and an inverse Fourier transformation for both water-suppressed and unsuppressed MRS data. Artifacts present in the time domain water-suppressed signal due to static magnetic field inhomogeneities and time-varying gradients were corrected by dividing the water-suppressed MRS signal by the non-water-suppressed signal (19), a procedure that does not affect relative signal intensities.

### Statistics

The NAA/Cr and NAA/Cho ratios resulted from the right and left thalamus in IGE patients and control group were calculated and compared using independent *t*-test in the IBM SPSS software version 11.5 (SPSS Inc., Chicago, IL, USA). Statistical significance was assumed as *P*-values < 0.05.

## Results

**Figure 2:**
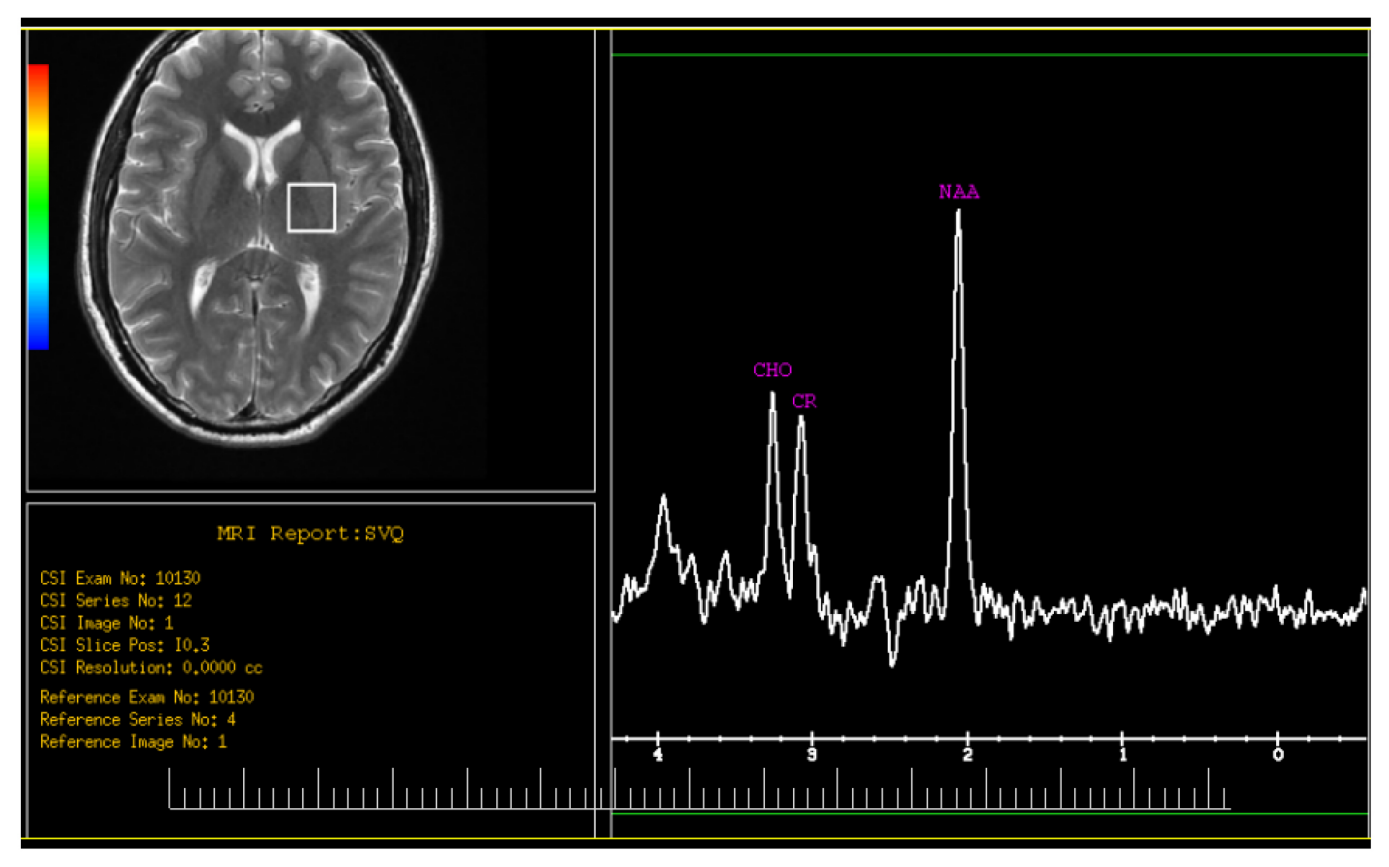
MRS spectra from a left side of thalamus in IGE patient

Thalamus metabolites including NAA, Cr and Cho were measured in both sides in IGE patients and control group. Mean and standard deviation (SD) values, the comparison of these metabolites, and NAA/Cr and NAA/Cho ratios are shown in Table 2. A sample of the MRS spectrum obtained from a patient is illustrated in Figure 2. The statistical tests showed that all of the metabolites did not have any significant differences between the right and left side of thalamus inside the groups. NAAs have significant lower values in IGE patients compared to healthy group (*P*-value = 0.01). The Cr values in the left side of thalamus showed remarkable higher values in IGE patients (*P*-value = 0.032); however, there was no difference in Cr values in right thalamus between IGE patients and healthy individuals. Amount of Cho showed no difference between the groups in both sides of thalamus.

**Figure 3:**
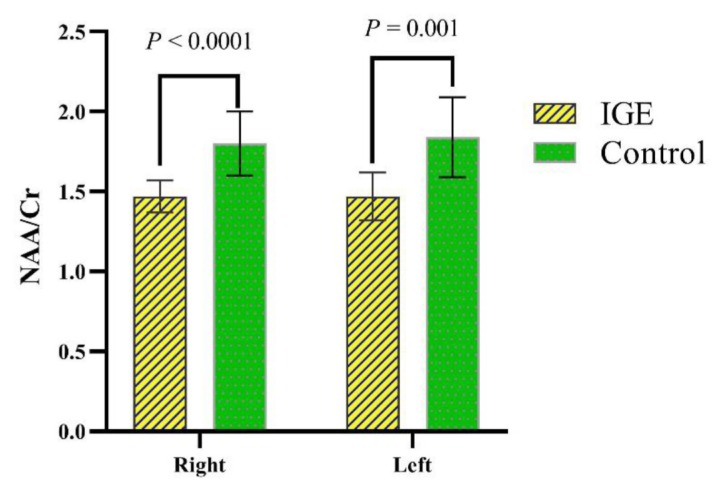
Comparison of NAA/Cr mean ratios in thalamus between IGE patients and control groups (*P*-values resulted from the *t*-test were shown above the compared groups. Error bars represent the standard deviations)

The SD values of the NAA/Cr ratios in the right and left thalamus in both IGE and healthy participants were measured and presented in Table 2 and Figure 3. The ratio of NAA/Cr among IGE patients was significantly lower than the healthy individuals (*P*-value ≤ 0.001). The average NAA/Cr ratio in the right and left thalamus in healthy participants were higher in comparison with those sides of thalamus in IGE patients.

**Figure 4:**
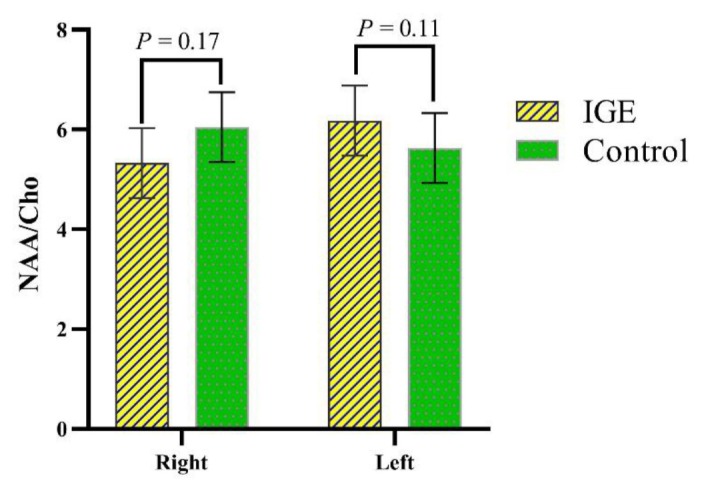
Comparison of NAA/Cho mean ratios in thalamus between IGE patients and control groups (*P*-values resulted from the *t*-test were shown above the compared groups. Error bars represent the standard deviations)

This study also evaluated the NAA/Cho ratios in the right and left thalamus. The results of the study showed that there was no significant difference between the NAA/Cho ratios in the IGE patients and control groups (Table 2 and Figure 4).

## Discussion

We found that changes in thalamus metabolites (NAA, Cr and NAA/Cr values) are the appropriate diagnostic biomarkers for differentiating IGE patients from healthy individuals using MRS technique.

In a study by Savic et al. (18) using a single-voxel spectroscopy (SVS) method, it showed that the NAA value in thalamus of idiopathic patients was lower compared with healthy people (9.7 [SD = 1.0] versus 10.8 [SD = 0.9]). In that study, they used a SVS method and did not assess the metabolites ratios like NAA/Cr.

In the current study, the multi-voxel methodology was used and the metabolite ratios in both side of thalamus were measured. In SVS technique, a single spectrum was acquired from a definite volume of tissue as an integral (12, 15, 17). Although this technique is fast and a spectrum is easily acquired, it provides low information about the organs’ metabolites (15). By contrast, the multi-voxel technique combining features of MRI and MRS data from multiple adjacent voxels covering a big volume of an organ (15).

The results of our study demonstrated that the value of NAA and NAA/Cr in the thalamus of IGE patients is significantly lower (*P*-value < 0.01) compared to healthy individuals. Since NAA is measured in a voxel, a reduction in NAA reflects a loss of nerve cells or axons, as well as neural damage and a disorder in body metabolism (11).

In this study, some evidence was found about the relationship between the metabolic changes and neural activity of thalamus in IGE patients as the main foundation of seizure generation in this type of epilepsy. Nevertheless, it was not determined whether the thalamus functioning disorder is specific to IGE or not. This is because in general, no structural damage was found in routine MRI images as a foundational area for epilepsy focus. The analysis of the spectrogram obtained through multi-voxel MRS method showed a remarkable decrease in the NAA and NAA/Cr ratio in the thalamus of IGE patients. We also found that the Cr value was higher in IGE patients in comparison with healthy individuals just in the left side of thalamus. This metabolite usually was used as internal reference, since it is the most stable cerebral metabolite. Therefore, higher values of Cr in IGE patients can indicate the neural problems. Previous studies (11, 12, 16) focused on the changes in NAA and NAA/Cr values for IGE patient thalamus; therefore, it is proposed to study the changes of thalamus Cr values in IGE patients in future researches.

In a study, Fojtiková et al. (16) reported that thalamic NAA/Cr ratio is significantly lower in patients with typical absence epilepsy in comparison with healthy group. In another study by Bernasconi et al. (11), the mean value of thalamic NAA/Cr in IGE patients group was lower compared to control healthy individuals. There was no difference in NAA/Cr between patients whose seizures were well controlled and those not controlled. They expressed that the thalamic dysfunction may occur regardless of amount of wave and spike activity. Doelken et al. (15) reported that NAA and Glx (glutamate + glutamine) metabolite values differ in IGE patients compared to healthy people. We found the similar results in our study. Furthermore, we evaluated the NAA and Cr values and a significant difference between the groups was obtained. The Glx or other metabolites were not evaluated because these metabolites have lower peaks in comparison with NAA, Cr and Cho peak values. The MR spectrum is along with noise and random errors, therefore, measuring high peak values in MRS like NAA, Cr, and Cho are less affected by noises and errors (20, 21).

According to the findings of this study and matching them with the clinical data of patients, thalamus plays a key role in the generation of seizures in IGE which means the presence of thalamo-cortical anomalies as a pathophysiologic foundation for IGE.

In this study, different time and locations for gathering the MRS data were the limitations. Furthermore, differences in therapeutic modalities, like various pharmacotherapy programs, may affect the results of our study.

Regarding the results of this study and previous researches (11, 12, 16, 17), it is suggested that more studies have to be conducted with respect to using the MRS technique for diagnosing various types of epilepsy. In addition, similar studies could be performed on a bigger group of patients to prove the efficiency of the MRS in diagnosis of epilepsy.

## Conclusion

Our results demonstrated certain altered metabolite values in IGE patients compared to healthy individuals. Thalamic NAA, Cr and NAA/Cr ratio values showed significant differences in IGE patients. Therefore, MRS of thalamus in patients is suggested for differentiating the IGE patients from healthy individuals.

## Figures and Tables

**Table 1:** Individual demographics (*n* = 70)

Variables		IGE Patients (*n* = 35) *n* (%)	Control (*n* = 35) *n* (%)	Total *n* (%)
Age (years)^a^		32 (5.2)	31 (6.1)	31.5 (5.7)
Gender	Male	11 (31.4)	22 (62.9)	33 (47.1)
	Female	24 (68.6)	13 (37.1)	37 (52.9)

Note:

a mean (SD)

**Table 2:** Comparison of NAA, Cr and Cho values, and also NAA/Cr and NAA/Cho ratios in both sides of thalamus between IGE patients and control groups

Variable	Mean (SD)		Mean differences (95% CI)	*P*-value
	Right thalamus of patients (*n* = 35)	Right thalamus of control (*n* = 35)		
NAA^a^ (mM/L)	9.6 (0.8)	10.9 (0.9)	1.3 (1, 1.61)	0.01
Cr^b^ (mM/L)	6.5 (0.6)	6.0 (0.7)	0.5 (0.32, 0.73)	0.12
Cho^c^ (mM/L)	1.8 (0.4)	1.8 (0.4)	0.1 (0, 0.11)	0.45
NAA/Cr	1.47 (0.11)	1.70 (0.20)	0.34 (0.25, 0.43)	< 0.0001
NAA/Cho	5.33 (0.71)	6.05 (0.70)	0.71 (0.53, 0.89)	0.17

	**Left thalamus of patients (*n* = 35)**	**Left thalamus of control (*n* = 35)**	**Mean differences (95% CI)**	***P*-value**

NAA^a^ (mM/L)	9.9 (0.7)	10.7 (0.9)	0.8 (0.4, 0.95)	0.01
Cr^b^ (mM/L)	6.7 (0.8)	5.8 (0.5)	1.2 (0.92, 1.51)	0.032
Cho^c^ (mM/L)	1.6 (0.3)	1.9 (0.6)	0.3 (0.21, 0.42)	0.23
NAA/Cr	1.47 (0.15)	1.84 (0.25)	0.38 (0.28, 0.49)	0.001
NAA/Cho	6.18 (0.72)	5.63 (0.70)	0.55 (0.41, 0.70)	0.11

Notes:

a N-acetylaspartat;

b Cr;

c Cho
